# Genome Sequence of *Leuconostoc mesenteroides* LK-151 Isolated from a Japanese Sake Cellar as a High Producer of d-Amino Acids

**DOI:** 10.1128/genomeA.00661-17

**Published:** 2017-07-27

**Authors:** Shiro Kato, Tadao Oikawa

**Affiliations:** a High Technology Research Core, Kansai University, Suita, Osaka, Japan; b Faculty of Chemistry, Materials, and Bioengineering, Kansai University, Suita, Osaka, Japan

## Abstract

Here, we report the complete genome sequence of strain LK-151 of *Leuconostoc mesenteroides*, which was isolated from a Japanese sake cellar and has the potential to produce large amounts of d-amino acids, namely, d-Ala and d-Glu. The genome contains 4 genes related to d-amino acid production.

## GENOME ANNOUNCEMENT

In general, the tastes of d-amino acids are quite different from those of the corresponding l-amino acids. Most of the d-amino acids taste sweet, and the threshold values of d-amino acids are almost in the same order as those of the corresponding l-amino acids (1, 2). Our recent study showed that there was a positive correlation between d-amino acid contents and the taste of Japanese sake (3). The d-amino acid analysis revealed that sake brewed with “kimoto,” a traditional method for brewing sake, tended to contain a high concentration of d-amino acids (4). In the kimoto method, lactic acid bacteria act as an important factor in killing contaminated bacteria. It has also been suggested that lactic acid bacteria might contribute to the production of d-amino acids during fermentation for other fermented foods, such as wine (5) and vinegar (6). These reports led us to the hypothesis that elucidation of the d-amino acid production mechanism of lactic acid bacteria may shed light on the origin of d-amino acids in fermented foods, and in this study, we analyzed the genome sequence of *Leuconostoc mesenteroides* strain LK-151, which was isolated from a Japanese sake cellar as a high producer of two d-amino acids, d-Ala and d-Glu (7).

The LK-151 genome was sequenced from DNA libraries prepared using a GS Titanium rapid library preparation kit and GS Titanium libraries of paired-end adaptors. The whole-genome shotgun reads and 8-kb-span paired-end reads from a GS Junior 454 sequencer (Roche) were assembled using GS De Novo assembler v. 2.9, yielding 1 scaffold with approximately 59-fold genome coverage. The remaining gaps were filled by Sanger sequencing, and final assembly revealed that the LK-151 genome comprises one chromosome of 2,090,103 bp and three plasmids (30,355, 14,042, and 2,869 bp) with 37.75%, 34.76%, 35.05%, and 35.48% GC content, respectively. The chromosome and 3 plasmids were predicted to contain 2,076, 33, 11, and 5 protein-coding genes, respectively. In addition, based on annotation from the Microbial Genome Annotation Pipeline (8), 68 tRNA and 9 rRNA genes were identified on the chromosome.

Using the KEGG automatic annotation server (9), we analyzed the putative d-amino acid metabolic pathway of strain LK-151. No genes coding for putative d-amino acid-degrading enzymes were detected. In terms of d-amino acid synthesis, 4 putative amino acid racemase genes, 3 alanine racemase homolog genes, and 1 glutamate racemase homolog gene were identified. The number of genes and their locations on the chromosome were identical to those of *L. mesenteroides* ATCC 8293, whose genome has been sequenced (10). *L. mesenteroides* NBRC 102480 also has the same genes, and the gene products have been revealed as alanine, lysine, histidine, and glutamate racemases (7), which is consistent with the d-amino acid-producing ability of strain LK-151. The high d-amino acid-producing potential of strain LK-151 might be due to the difference in transcriptional and flux levels.

### Accession number(s).

The complete genome sequence has been deposited in DDBJ under the GenBank accession numbers AP017936 (chromosome), AP017937 (plasmid), AP017938 (plasmid), and AP017939 (plasmid).
